# *Sleeping Beauty* Transposon-Mediated Asparaginase Gene Delivery by a Nanoparticle Platform

**DOI:** 10.1038/s41598-019-47927-6

**Published:** 2019-08-07

**Authors:** Jen-Hsuan Chang, Kurt Yun Mou, Chung-Yuan Mou

**Affiliations:** 1 Department of Chemistry, National Taiwan University, Taipei, 106 Taiwan; 2 Institute of Biomedical Sciences, Academia Sinica, Taipei, 11529 Taiwan; 3 Graduate Institute of Nanomedicine and Medical Engineering, Taipei Medical University, No. 250, Wu Xinyi Street, Taipei, 11031 Taiwan

**Keywords:** Transfection, Drug delivery

## Abstract

Transgenic genome integration using non-viral vehicles is a promising approach for gene therapy. Previous studies reported that asparagine is a key regulator of cancer cell amino acid homeostasis, anabolic metabolism and cell proliferation. The depletion of asparagine would inhibit the growth of many cancer cells. In this study, we develop a nanoparticle delivery system to permanently integrate the asparaginase gene into the genome of human lung adenocarcinoma cells. The asparaginase plasmid and the *Sleeping Beauty* plasmid were co-transfected using amine-functionalized mesoporous nanoparticles into the human lung adenocarcinoma cells. The intracellular asparaginase expression led to the cell cytotoxicity for PC9 and A549 cells. In addition, the combination of the chemotherapy and the asparaginase gene therapy additively enhanced the cell cytotoxicity of PC9 and A549 cells to 69% and 63%, respectively. Finally, we showed that the stable cell clones were successfully made by puromycin selection. The doxycycline-induced expression of asparaginase caused almost complete cell death of PC9 and A549 asparaginase-integrated stable cells. This work demonstrates that silica-based nanoparticles have great potential in gene delivery for therapeutic purposes.

## Introduction

Non-viral gene therapy vectors are being studied intensively in human gene therapy due to the simplicity of construction, customized individual needs and lower costs of production compared to viral gene therapy vectors^1,2^. The *Sleeping Beauty* (SB) transposon system^3^ is a non-viral vector that can mediate stable integration of therapeutic transgenes into the genomes of treated cells^4,5^ and provides sustained expression over a long time. Gene therapy based on SB has the potential to become an effective component of cancer treatment by transferring genes that cause tumor cell death or that inhibit angiogenesis^4^. The major obstacle to using non-viral vectors *in vivo* is the delivery to target cancer cells because naked DNA has difficulty in cellular uptakes and tumor targeting^6,7^. A nanocarrier system for the delivery gene into the specified tumor for cancer therapy would be very desirable for overcoming these barriers^8^.

Enzymatic therapy has been developed for the treatment of tumors^9,10^. Asparagine, a semi-essential amino acid in humans, is crucial for the growth of human cancers, and it plays an important role in tumor metabolism^11,12^. The tumor cells would undergo cell apoptosis when glutamine-dependent asparagine synthesis was suppressed^13,14^. The asparaginase synthetase is widely expressed in eukaryotic cells, but it is absent or low expressed in several cancer cells, for example, the acute lymphoblastic leukemia^15,16^. Therefore, enzymatic depletion of asparagine is a promising approach for cancer therapy^17,18^. Avramis and Tiwari reported that native and PEGylated L-asparaginase could deaminate L-asparagine into aspartic acid and ammonia, killing T-lymphoblastic leukemia^19–21^. Zhang *et al*. demonstrated that the depletion of asparagine by asparaginase could induce remarkable cytotoxicity and apoptosis in human lung adenocarcinoma cells^22^. Note that the most widely utilized asparaginase from *E*. *coli* also processes a weak glutaminase activity^23^, which may also contribute to the cancer elimination^24^. Likewise, other non-essential amino acids could also be targets of depletion. Arginine depletion was used for the treatment of breast cancer^25,26^. Savaraj *et al*. studied that arginine deiminase (ADI) would lead to the degradation of arginine resulting in cell apoptosis of melanoma^27^.

Limitations arise from the difficulty of delivering enzymes, including asparaginase, to cells^28^. Poor stability of the protein, the subsequent immune response, endosome trapping, and protein degradation are all problematic issues that are difficult to be addressed in enzyme delivery, limiting its clinical use to liquid cancers^29^. Furthermore, periodical delivery of enzymes could be expensive. A better approach would be a gene delivery by nanocarrier to targeted cells that express the therapeutic protein inside or nearby (in the case of amino acid depletion) the targeted cells. An exciting recent example is using nanoparticle to carry *piggyBac* transposon system for *in situ* programming patient-derived T cells with genes encoding disease-specific chimeric antigen receptors (CARs) that target leukaemia^30^.

**Figure 1:**
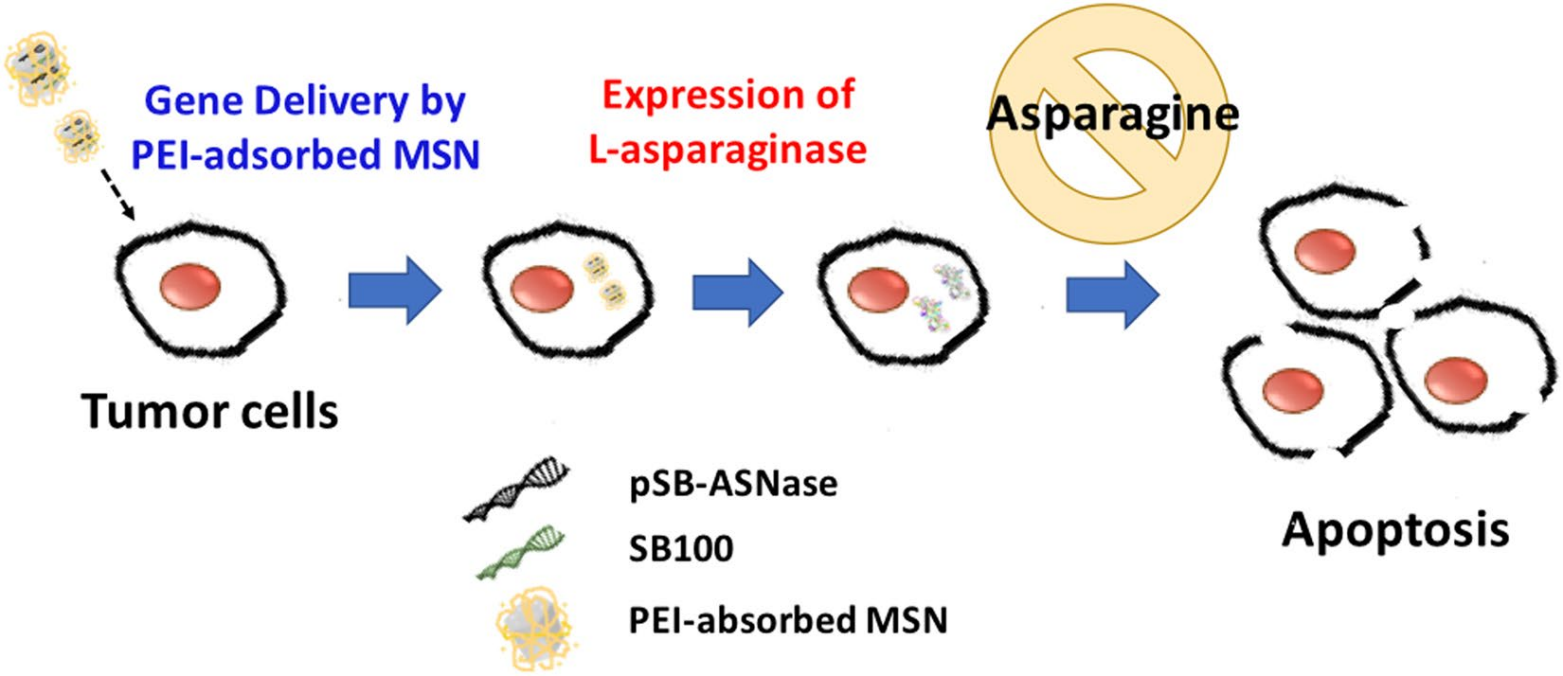
The nanoparticle delivery of the *Sleeping Beauty* transposon system to mediate the asparaginase (ASNase) gene integration into cancer cells. Two vectors, the transfer vector pSB-ASNase and the *Sleeping Beauty* vector SB100, were co-delivered by the PEI-absorbed mesoporous silica nanoparticles. The intracellular expression of asparaginase depletes the asparagine supply and causes the cell death.

Mesoporous silica nanoparticles (MSN) is a good nanocarriers with its ease of surface functionalization, high surface area (>1000 m^2^g^−1^) and tunable pore sizes (1.5–10 nm). In addition, MSN is non-toxic and have been widely applied to delivery systems^27–30^. With further functionalization of PEI, endosomal escape of MSN can be enhanced by proton sponge effect. Herein, we developed the first non-viral gene delivery for asparaginase expression using the SB transposon vectors by polyethyleneimine (PEI)-absorbed MSN to induce lung cancer cell apoptosis (Fig. 1). The SB system could efficiently integrate the target gene into the host chromosome for long-term expression both *in vitro* and *in vivo*^31–36^. Prior to this study, our lab has successfully accomplished the transient gene delivery using MSN into induced pluripotent stem cells for specific cell-oriented differentiation^37,38^. While transient transfections are ideal for certain experimental settings, stable cell lines could be a more reliable approach for long-term observation as well as for *in vivo* implantation. In this study, we used MSN to deliver the SB transposon plasmids and successfully created stable cell lines expressing the asparaginase. The intracellular expression of asparaginase caused significant cell death in two lung cancer adenocarcinoma cells, PC9 and A549. In addition, we found that the asparaginase gene therapy is additive to the common chemotherapy. We expect that the MSN-delivered transposon system could be applied *in vivo* for targeted gene therapy in the future.

## Result and Discussion

### Characterization of amine-modified mesoporous silica nanoparticles (MSN-NH_2_)

**Figure 2:**
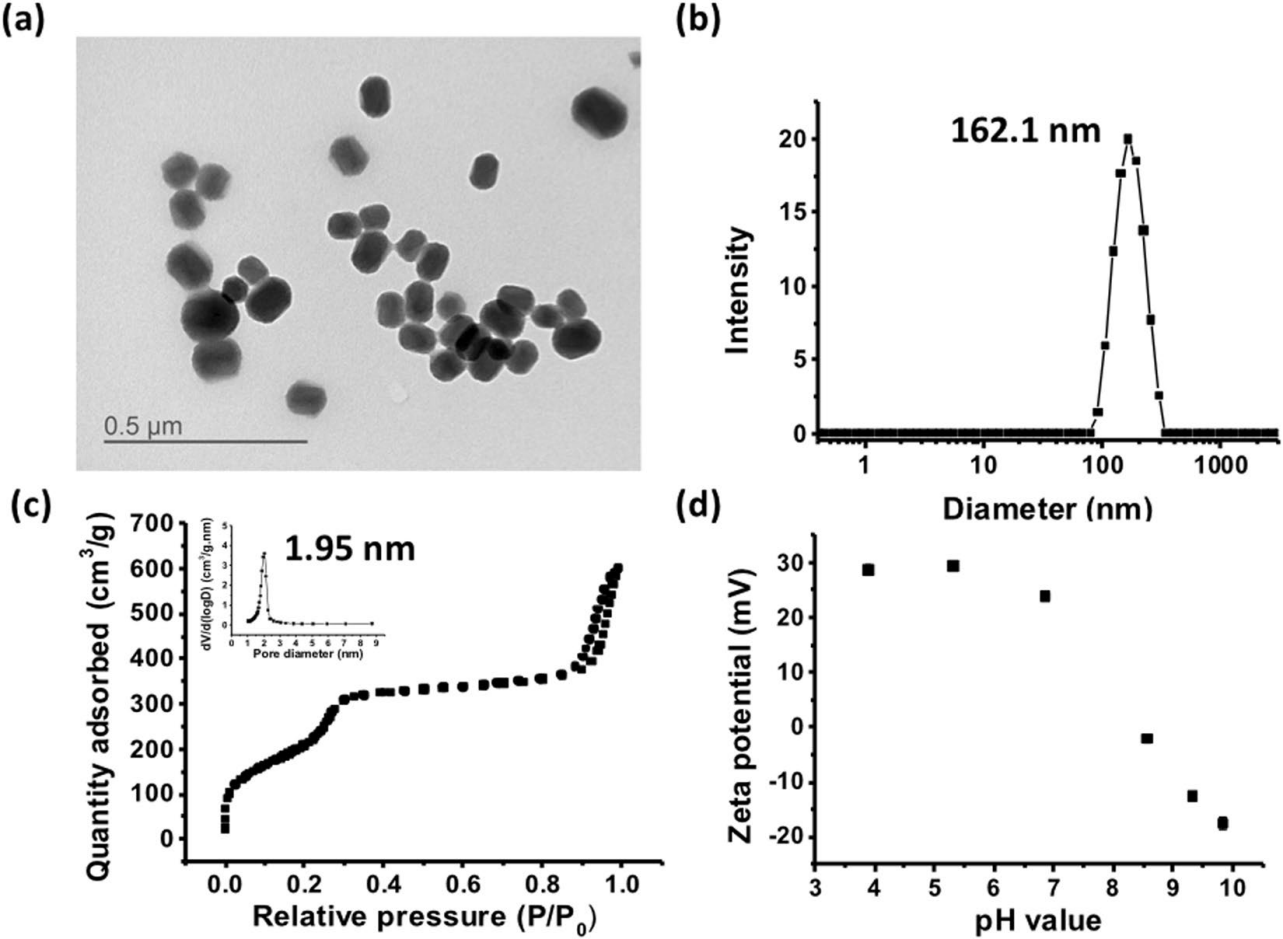
The amine-modified mesoporous silica nanoparticles (MSN-NH_2_) is homogeneous and well-dispersed in the water solution. (**a**) Representative TEM of MSN-NH_2_, (**b**) DLS size measurements of MSN-NH_2_, (**c**) N_2_ adsorption-desorption isotherm of MSN-NH_2_ and (**d**) The pH-dependent zeta potentials of MSN-NH_2_.

MSNs were synthesized by base catalyzed sol-gel reaction with cetyltrimethylammonium bromide (CTAB) as templates, and 3-aminopropyltriethoxysilane (APTMS) was used to functionalize MSNs into amine group-functionalized nanoparticles (abbreviated as MSN-NH_2_). A representative transmission electron microscopy (TEM) image of MSN-NH_2_ is shown in Fig. 2a. Based on the TEM image, the size of MSN-NH_2_ is 92.9 ± 15.7 nm with an oval shape. The dynamic light scattering (DLS) showed a comparable particle size of 162.1 nm (Fig. 2b). The N_2_ adsorption-desorption isotherm is shown in Fig. 2c. The pore size determined by Barrett-Joyner-Halenda (BJH) analysis is 1.95 nm. The internal pores will be used for carrying tracking fluorescence agents or other small molecule drugs such that they do not interfere with the carrying of the plasmid. The Brunauer–Emmett–Teller (BET) surface area is 766.47 m^2^ g^−1^. Figure 2d shows the pH-dependent zeta potential of MSN-NH_2_. Under the physiological condition, the MSN-NH_2_ particles are positively charged owing to the amine functionalization. The amination weighs 14.7 ± 0.2% of the mesoporous silica nanoparticles as determined by thermogravimetric analysis (TGA) (Fig. S2). The PEI was physically adsorbed on the MSN-NH_2_. The amount of PEI adsorption was determined to be 18.0 ± 1.1% by TGA. The resulting material PEI-adsorbed MSN showed no apparent aggregation with a particle size of ~180–190 nm (Table S2). The zeta potential at pH = 7.4 was increased up to +40.9 due to the PEI adsorption. Further complexation of PEI-adsorbed MSN and the SB plasmids (pSB-ASNase + SB100) caused no significant aggregations (208.0 nm) to slight aggregations (447.1 nm) when increasing the dose of PEI from 2.25 μg to 4.5 μg (Table S2).

### PEI-adsorbed MSN showed good uptake and low cytotoxicity to human lung cancer cells

**Figure 3:**
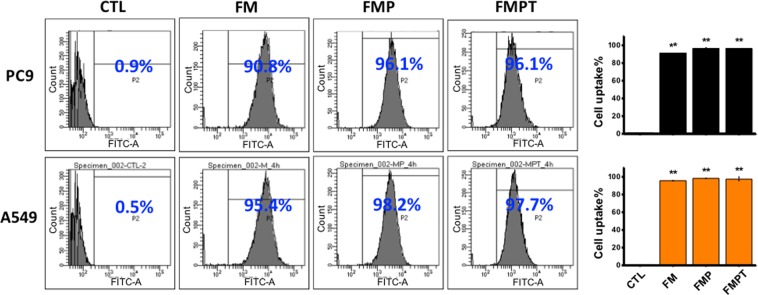
PC9 and A549 showed great cellular uptake of the amine-modified mesoporous silica nanoparticles within 4-hour incubation. FM: FITC-conjugated MSN-NH_2_; FMP: PEI-absorbed FMSN; FMPT: T + PEI-absorbed FMST; T: pSB-ASNase + SB100. The amount of FMSN was 25 µg/mL, the PEI was 2.25 µg/mL for PC9 cells and 4.5 µg/mL for A549 cells. T: pSB-ASNase + SB100. **Indicates the statistical differences (**p < 0.001) compared with controls.

For efficient gene delivery, a good cell uptake of nanoparticles with high endosomal escape is critical. We used flow cytometry to quantify the cellular uptake efficiency of the nanoparticles. All particles tested (FM, FMP, and FMPT) showed great cell uptake with the efficiency over 90% after 4-hour incubation (Fig. 3). We then used confocal microscopy to visualize the endosomal escape of nanoparticles in PC9 and A549 cells at various time points (Fig. S3). At 15 min, PEI-adsorbed FMSN were aggregated around the cell membrane. The nanoparticles co-localized with an early endosome marker EEA1 after 30 min. Co-localization decreased after 60 min, suggesting that the nanoparticles successfully escaped from the endosomes and resided in the cytoplasm.

**Figure 4:**
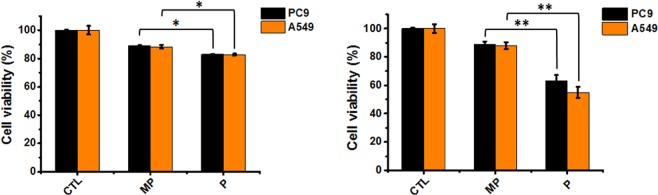
The PEI-absorbed MSN significantly reduced the cytotoxicity of free PEI in PC9 and A549 cells. MP: PEI-absorbed MSN; P: free PEI. The PC9 and A549 cell viability was measured after the treatment of PEI and PEI-absorbed MSN (PEI, 2.25 µg/mL for PC9 cells, 4.5 µg/mL for A549 cells, MSN: 25 µg/mL) for (**a**) 24 hr and (**b**) 48 hr. *And **indicates the statistical differences (*p < 0.05 and **p < 0.001) compared with controls.

We further evaluated the cell cytotoxicity caused by the nanoparticles. PC9 and A549 cells were treated with PEI or PEI-absorbed MSN, and the cell viability was measured after 24 hr and 48 hr. As expected, the free PEI is cytotoxic to the cells with the viability down to ~60% after 48 hr of the treatment (Fig. 4). Interestingly, the PEI-adsorbed MSN treatment significantly improved the viability to more than 85% for both PC9 and A549 cells. This experiment implicates that the PEI was tightly adsorbed on the MSN-NH_2_ particles, therefore sequestering the cytotoxicity of PEI.

### PEI-absorbed MSN successfully delivered the *Sleeping Beauty* system for asparaginase expression

**Figure 5:**
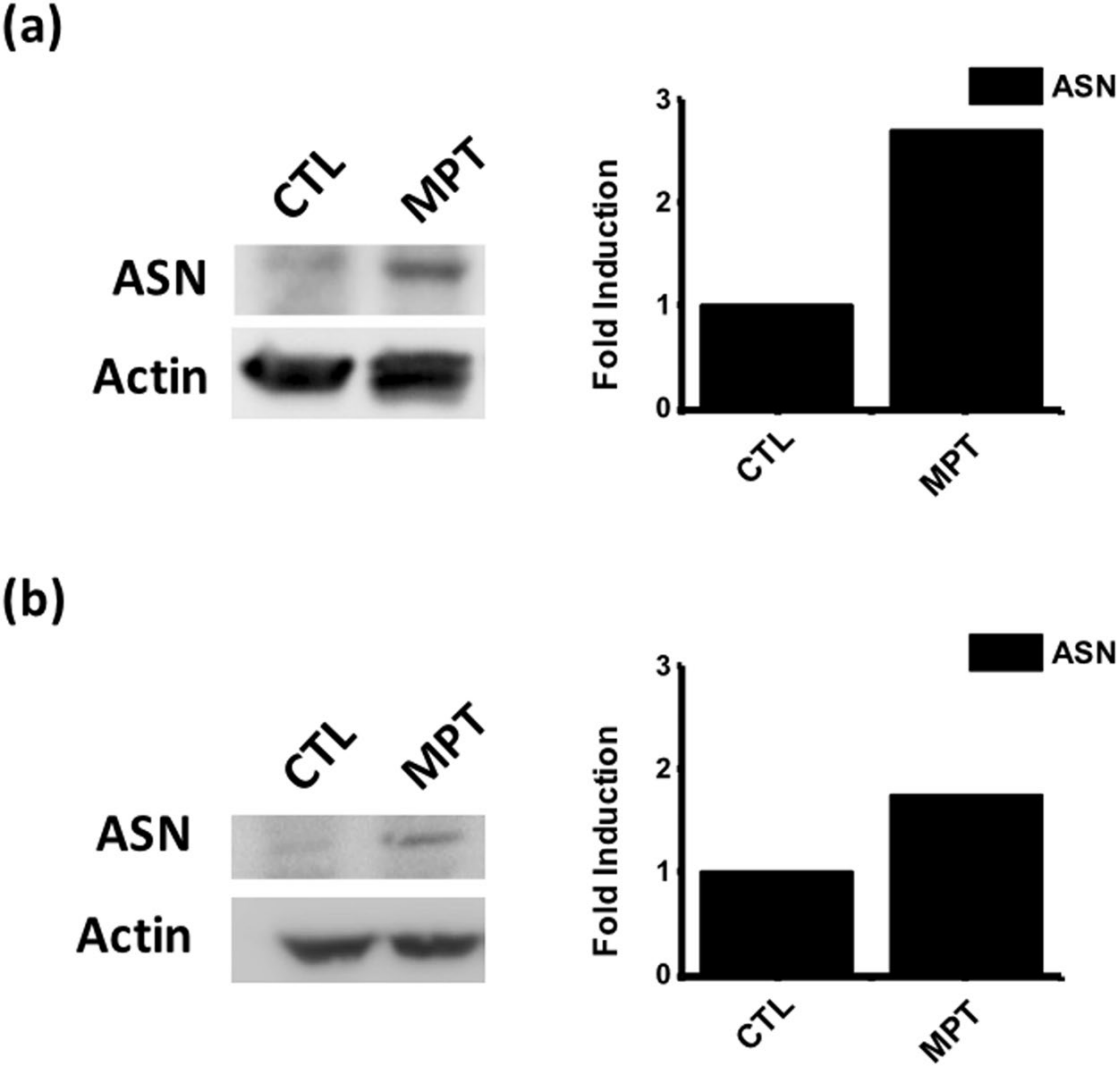
The asparaginase gene was successfully delivered and expressed by the PEI-absorbed MSN transfection in PC9 and A549 cells. MPT: T + PEI-absorbed MSN (T: pSB-ASNase + SB100). (**a**) PC9 cells, and (**b**) A549 cells under the treatment of MPT or the non-treatment condition (CTL) for 4 days.

We next sought to analyze the transfection efficiency using the MSN nanoparticles. We co-delivered the SB plasmids pSB-ASNase and SB100, where the pSB-ASNase plasmid encodes a doxycycline-inducible asparagine gene and a green fluorescent protein (GFP) reporter gene. The GFP-positive cells were determined by flow cytometry after 2 days of transfection. Figure S4 showed that the transfection efficiencies were 2.4 ± 0.2% and 3.3 ± 0.9% for PC9 cells and A549 cell, respectively, when using MSN-NH_2_ particles alone. The transfection efficiency was improved to 8.8 ± 0.1% and 7.7 ± 0.0%, respectively, when PEI-absorbed MSN particles were used. Note that the improvement was due to the PEI absorbed on the particles as the cytotoxicity from the free PEI was not observed. We have also used two widely utilized commercial transfection reagents PEI and Lipofectamine 3000, which exhibited inferior efficiencies around 3–6% compared to the PEI-absorbed MSN. Note that the Lipofectamine 3000 transfection was performed according to the vendor’s protocol. The suboptimal transfection efficiency might be improved by further optimization. For the asparagine quantification, we added doxycycline to induce the asparagine expression after one day of transfection and incubated for 3 days. We quantified the asparaginase expression level by western blot. Figure 5 showed that the asparaginase expression levels in PC9 and A549 cells were higher than the negative controls by 2.5 and 1.8 folds. The successful asparaginase induction was also validated by qPCR at the mRNA level (Fig. S5).

### Genetic delivery of asparaginase is effective for cancer therapy and additive with chemotherapy

**Figure 6:**
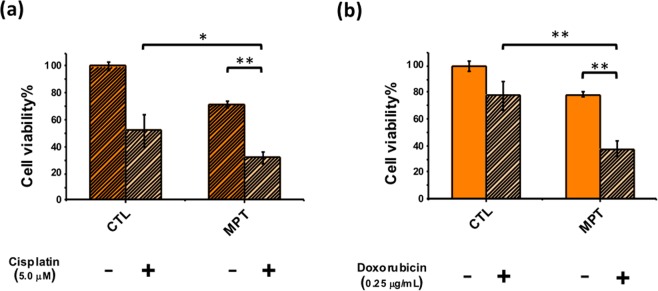
The cytotoxicity of the asparaginase gene expression and the chemotherapy is additive in PC9 and A549 cells. The cell proliferation assays were demonstrated under the treatments of (**a**) cisplatin (5.0 µM) in PC9 cells and (**b**) doxorubicin (0.25 µg/mL) in A549 cells after the transfection of pSB-ASNase and SB100. *p < 0.05 and **p < 0.001. MPT: T + PEI-absorbed MSN (T: pSB-ASNase and SB100).

Previous studies have shown that asparagine was involved in coordinating protein and nucleotide synthesis in certain cancer cells. Consequently, their survival and growth are fully dependent on the external supply of asparagine. Therefore, the asparagine depletion therapy, such as asparaginase, has proved useful for treating certain malignancies. We first showed that two human lung adenocarcinoma cell lines, PC9 and A549, are sensitive to the asparaginase treatment with IC50 4 unit/mL and 3 unit/mL, respectively (Fig. S6). Two facts should be noted here: (i) PC9 and A549 are competent to *de novo* synthesize asparagine; (ii) the *E*. *coli* asparaginase used here has weak glutaminase activity (asparaginase activity/glutaminase activity ~500:1^23^). The exogenous asparaginase cytotoxicity could be due to an additive effect of both asparagine and glutamine depletions. Next, we sought to use the nanoparticle vehicle to deliver the asparaginase gene with a doxycycline-inducible SB system. The *Sleeping Beauty* plasmids pSB-ASNase and SB100 were co-delivered by the PEI-absorbed MSN particles (MSN + PEI + pSB-ASNase, abbreviated as MPT). After one day of transfection, doxycycline was added to induce the asparagine expression in the cells. The cell viability was examined after 48 hours of transfection. Note that we have tested that the doxycycline treatment alone is not cytotoxic to PC9 or A549 cells (Fig. S7). As shown in Fig. 6, the viability of the MPT-transfected cells decreased approximately 28 ± 2.6% in PC9 cells and 22 ± 1.6% in A549 compared to the untreated cells. Note that 10% PC9 and 7% A549 cell death were found for the PEI-absorbed MSN treatment alone (data not shown). Furthermore, we tested if the asparaginase gene therapy and the chemotherapy are additive. We used cisplatin or doxorubicin co-treated with the MPT. After the MPT transfection for two days, 5 µM of cisplatin or 0.25 µg/mL of doxorubicin was added to the culture medium. The cell viability assay was performed after 2 days. Figure 6 showed that the cisplatin treatment alone led to 48 ± 11.9% cell death of PC9, whereas the cisplatin and MPT co-treatment increased the death rate to 69 ± 4.3%. Similarly, the doxorubicin treatment alone caused only 2 2 ± 10.7% of A549 cell death, whereas the doxorubicin and MPT co-treatment greatly improved the cell death to 63 ± 5.6%. The data suggested that our nanoparticles could successfully deliver the asparaginase gene as a potent therapy to the asparagine-sensitive cancer cells, especially when co-treated with chemotherapy.

### Nanoparticles successfully delivered the *Sleeping Beauty* system to create the ASNase-carried stable cell line

**Figure 7:**
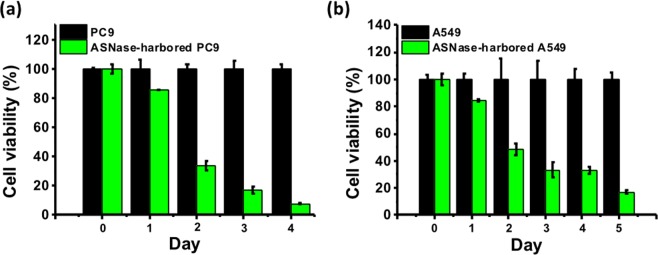
The ASNase-carried stable cell lines PC9 and A549 showed severe cell death after inducing the asparaginase expression. (**a**) After the asparaginase induction by doxycycline, the cell viability of the ASNase-carried stable cell line PC9 (PC9-MPT) was measured from day 0 to day 4. The signal was normalized to the parental PC9 cells. (**b**) The same experiment for A549.

Next, we sought to test if the *Sleeping Beauty* system delivered by the nanoparticles could successfully create stable cell lines. The pSB-ASNase plasmid encoded a puromycin resistance gene that could be used for stable cell line selections. After co-transfection of pSB-ASNase and SB100 by PEI-adsorbed MSN for 2 days, we challenged the PC9 and A549 cells with 0.5 µg/mL puromycin for 7 days. All the survived cells of PC9 and A549 (ASNase-carried PC9 and ASNase-carried A549) showed clear GFP signals as shown by fluorescence microscopy (Fig. S8). Continuous growth of the cells in the absence of puromycin maintained the GFP expression, indicating a stable integration of pSB-ASNase into the host genome by the *Sleeping Beauty* system. To investigate the full strength of the asparaginase gene therapy, we performed the asparagine depletion experiments with the ASNase-carried PC9 and ASNase-carried A549 stable cells. The time-course cell viability was measured after the asparaginase induction by doxycycline (Fig. 7). The cell viability was dropped to only 7.8% and 17.1% for the ASNase-carried PC9 cells on day 4 and the ASNase-carried A549 cells on day 5, respectively. The ASNase-carried PC9 or A549 cells also showed much higher sensitivity to the exogenous asparaginase treatment compared to the parental PC9 or A549 cells (Fig. S9). Note that the parental PC9 or A549 cells were also sensitive to the asparaginase treatment when growing in the asparagine-free DMEM medium, which likely reflected the cytotoxicity caused by the glutaminase activity from the asparaginase (Fig. S9). Interestingly, the RPMI-adapted ASNase-carried PC9 or A549 are more sensitive to the asparaginase treatment than the DMEM-adapted PC9 or A549. We reasoned that the growth of the RPMI-adapted cells might rely more heavily on asparagine than the DMEM-adapted cells.

### ASNase-carried cells depleted the environmental asparagine and reduced the cell growth to the neighboring cells

**Figure 8:**
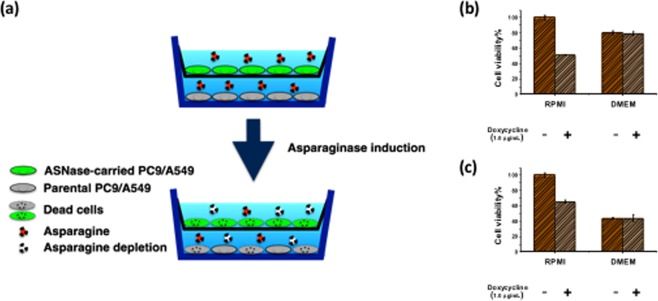
The ASNase-carried PC9 or A549 stable cells depleted the asparagine in the medium and caused the cytotoxicity to the co-cultured parental PC9 or A549 cells. (**a**) The experimental design of the transwell co-culture assay. The doxycycline-inducible ASNase-carried PC9 or A549 stable cells were seeded in the inner chamber, and the parental PC9 or A549 cells were seeded in the outer chamber. After 48 hours of the asparaginase induction by doxycycline, the cell viability was assayed for the parental cells in the outer chamber. (**b**) The cell viability was compared between the doxycycline-treated and the non-treated experiments for PC9 in RPMI or DMEM medium. (**c**) same as (**b**) for A549.

In the previous experiments, we noticed that 7–8% transfection efficiency could lead to 22–28% cell death for PC9 and A549 (Figs S4 and 6). Although a portion of cell death could be contributed by the transfection toxicity (<10%), we suspected that the transfected cells might cause additional cell death to the non-transfected cells through the asparagine depletion in the medium. To test this hypothesis, we designed a transwell experiment, in which the inner chamber was seeded with the ASNase-carried PC9 or A549 stable cells and the outer chamber was seeded with the parental PC9 or A549 cells (Fig. 8a). The cell viability of the parental cells in the outer chamber was compared between the conditions with or without the doxycycline induction after 48 hour. The doxycycline-treated experiments showed significant lower cell viability, which is only 50% and 60% of the non-treated experiments for PC9 and A549, respectively (Fig. 8b,c). This result supports our hypothesis that the asparaginase induction in the ASNase-carried cells not only kills themselves but also effects the viability of the co-cultured parental cells due to the asparagine depletion in the medium. Note that the PC9 and A549 cells can *de novo* synthesize asparagine. The asparagine depletion in the medium only slowed down the growth but not killed the cells. Expectedly, when performing the transwell experiment in DMEM, we observed no cytotoxicity to the outer chamber cells because DMEM is an asparagine-free medium (Fig. 8b,c). This neighboring effect is very encouraging for the future *in vivo* work because perfect transfection efficiency may not be required for effective tumor elimination.

## Conclusions

In summary, we have developed a mesoporous silica nanocarrier system that can deliver a *Sleeping Beauty* system, which endows a long-term expression of L-asparaginase for depletion of the amino acid asparagine. We demonstrated the asparaginase gene delivered by the nanoparticles could effectively kill two human lung adenocarcinoma cells, PC9 and A549, with great additive effect to common chemotherapy drugs. The nanocarrier system can be improved in several ways for *in vivo* studies in the future: (i) a surface functionalization of MSNs by cancer-targeting ligands can be easily executed by surface silane coupling reactions. Also, the MSN particles can be further functionalized with polyethylene glycol (PEG) to increase its blood circulation. (ii) the MSNs can be synthesized into specific sizes to accumulate in the tumor by enhanced permeability and retention (EPR) effect. The newly formed tumor has fenestrated blood vessel with hyper-permeability. The nanoparticle size is chosen such that they can take advantage of the enhanced permeability and retention (EPR) effect based on the hyper-permeability of tumor vessels, which allows certain sizes of nanoparticles to accumulate in the tumor. (iii) hollow silica nanospheres (HSN)^39^ may be used instead of MSNs for more elaborate loadings of internal cargos. Recently, Ma *et al*. improved the efficiency of a SB/carrier system by loading *in situ* PCR-amplified *Sleeping Beauty* transposon^36^.

A note about the amino acid depletion method in cancer therapy should also be mentioned here. Because some cancer cells down-regulated the synthesis of asparagines, they have to import asparagine from extracellular media. The delivery of our nanocarrier system for amino acid depletion needs only to the local environment of the cancer cell. Once asparagine molecules are efficiently removed from the local microenvironment of cancer, the cancer cells will die from starvation. This is especially suitable for our SB system since the expression of ASNase is localized to tumor cells and effective for long term.

Finally, we would like to comment that our nanocarrier/gene delivery platform may be useful for another major class of unmet medical need, e.g. the lysosomal storage disease (LSD), such as Fabry disease. LSDs are inherited diseases in which lysosomal enzymes are deficient and there is no cure^40^. Present approaches of enzyme replacement therapy (ERT) are very inefficient and expensive because of the biological barriers for enzyme delivery^41^. An approach of long-term production of enzymes intracellularly by the nanocarrier/SB system would be most desirable. The encouraging results of this report may make this goal closer.

## Experimental Section

### Synthesis of amine-group conjugated mesoporous silica nanoparticles

0.005 M of C_16_TAB (hexadecyltrimethyl ammonium bromide) was dissolved in 0.51 M of ammonia solution under constant agitation (30 min, 40 °C). Then, 5 mL of 0.21 M TEOS (tetraethoxysilane) ethanol solution was added with stirring for 1 hr at 40 °C, and then 5 mL of 0.88 M TEOS ethanol solution was added with stirring (30 min, 40 °C). Next, 400 μL of (3-aminopropyl)trimethoxysilane (APTMS) was added with stirring (30 min), and the solution was standing for 12 hrs. Then, 450 μL of PEG-silane (Mw 400) and 200 μL of ammonia solution (35%) was added with stirring (3 hr, 50 °C). The amine-functionalized MSN-NH_2_ was collected by centrifugation (15 min, 14000 rpm), washed, and redispersed with deionized water and ethanol several times. Next, the C_16_TAB was extracted by NH_4_NO_3_ (0.42 M of NH_4_NO_3_ ethanol solution) twice. Finally, the amine group-conjugated MSN-NH_2_ was collected by centrifugation (15 min, 14000 rpm).

### Labeling of RITC-pSB-ASNase

To study the plasmid release, the rhodamine isothiocyanate (RITC)-labeled pSB-ASNase was prepared. 200 μL of pSB-ASNase (0.5 μg pSB-ASNase/μL H_2_O) was mixed with 100 μL of RITC (10 mg RITC/mL EtOH). After the solution was stirred at 4 °C overnight, the unreacted RITC was removed by dialysis for three days and the RITC-pSB-ASNase was collected.

### Molecular cloning

The SB transfer plasmid (pSB) was digested with the restriction enzyme *SfiI*. The inserts were prepared by designing the PCR primers (Table S1) of asparaginase (ASNase) gene and using *Escherichia coli* cells as the template to amplify the DNA fragment of ASNase gene (Fig. S1). Next, the inserts were assembled with the digested pSB vector by the NEBuilder HiFi DNA Assembly Master Mix (NEB, E5520S). The final plasmid was sequencing verified and denoted as pSB-ASNase.

### Cell uptake analysis

The uptake efficiency of nanoparticles was determined by FACSCalibur flow cytometer and CellQuest Pro software (Becton Dickenson, Mississauga, CA). FITC-conjugated MSN (FMSN) were synthesized for detection of the fluorescence signal. The 25 µg/well FMSN and PEI-absorbed FMSN (PEI: 2.25 µg for PC9 cells and 4.5 µg for A549 cells) were incubated with cells (1 × 10^5^/well) for 4 hr and then washed by phosphate-buffered saline (PBS).

### Cellular cytotoxicity of PEI-absorbed MSN

1 × 10^4^ of PC9 or A549 cells per well were seeded in 24-well plates for 24 h before cytotoxicity assays. To evaluate the *in vitro* cytotoxicity of PEI and PEI-absorbed MSN, cells were incubated with PEI-absorbed MSN (4.5 μg of PEI mixed with 25 µg of MSN) in 24-well plates for 24 hr and 48 hr with regular growth medium. The cell was followed by the incubation of WST-1 (0.5 mg mL^−1^) for 2 hr at 37 °C for the cell cytotoxicity assay. The amount of orange formazan dye generated by the living cells was proportional to the number of the living cells, and the absorbance at 450 nm was measured by using a microplate reader (BioTek Synergy H1 Hybrid Reader).

### Cell culture, transfection, and cell selection

The human lung adenocarcinoma cells (PC9) and the human alveolar adenocarcinoma cell (A549) were maintained in RPMI 1640 supplemented with 2 mM glutamine, 10% Fetal Bovine Serum (FBS, GIBCO), 100 U/mL penicillin and 100 µg/mL streptomycin (GIBCO) at 37 °C in a 5% CO_2_ atmosphere. Cells were passaged every 3 days. Before transfection, the cells were seeded into 6-well dishes at a density of 1 × 10^5^ cells/well. 1.6 *μ*g of pSB-ASNase and 0.4 µg of SB100 plasmid were mixed with 25 µg of MSN for 30 mins, followed by addition of PEI for 10 mins (PEI, 2.25 µg for PC9 cells and 4.5 µg for A549 cells). The complexes were then added to cells and incubated for 4 hrs. After a wash step with PBS, the cells were cultured at 37 °C in RPMI 1640 supplemented with 10% FBS and 1 µg/mL doxycycline. After three days of transfections, 0.5 µg/mL of puromycin was used to select for 7 days to generate the stable cell clones.

### Immunofluorescence staining

Immunofluorescence staining was used to identify early endosome in PC9 cells. After FMSN incubated with PC9 cells for 15, 30 and 60 min, the PC9 cells were washed with PBS and fixed with 1% paraformaldehyde for 10 min. The PC9 cells were then permeabilized with 0.1% NP-40 for 20 min, and then the cells were subsequently incubated overnight with the early endosome marker EEA1 antibody (Abcam, ab70521). The cells were then washed with PBS and treated with the fluorescence-conjugated secondary antibody (Jakson Immunoreseatch, 111-545-003, 315-545-003) and DAPI. The stained PC9 cells were observed using a fluorescence microscope.

### Western blot

3 × 10^5^ of cells were seeded in 10 cm dishes and transfected with pSB-ASNase and SB100 using PEI-absorbed MSN for 4 hr. After 2 days, cells were trypsinized and lysed using 200 μL of M-PER^®^ Mammalian Protein Extraction Reagent (78501, Thermo Fisher) on ice for 30 min. The lysates were purified by centrifugation 1(4000 rpm, 15 min). Protein concentrations were quantified using a Pierce™ BCA Protein Assay Kit (23225, Thermo Fisher). The protein was denatured at 100 °C for 10 min and loaded into the SDS-PAGE gel. Proteins were transferred using the TE22 Mighty Small Transfer Tank (Hoefer) onto PVDF membranes (Millipore, IPVH00010). The membrane was blocked with 5% milk for 1 hr followed by the primary antibody (1:1000 of anti-ASNase (Abcam, ab55824), 1:5000 of anti-Actin (Millipore, MAB1501)) diluted in 5% milk followed by 1:5000 of HRP-conjugated secondary antibody (1:5000 dilution of Cell signaling, #7074, #7076). The signal was detected by adding the ECL-Plus Western Blotting substrate and a luminescence imaging system.

### Transwell co-culture experiments

The ASNase-carried PC9 or A549 cells were co-cultured with the parental PC9 or A549 cells in a transwell setup (Falcon Cell Culture Insert, #353091, 6-well, pore size 3 μm). A number of 50,000 ASNase-carried and parental cells were seeded in the inner chamber and the outer chamber, respectively, in a six-well plate. After 24 hours, 1 μg/mL doxycycline was added to the medium. A control experiment without the doxycycline treatment was also performed. After 48 hours of the doxycycline treatment, the cell viability was measured for the parental cells in the outer chamber by the WST assay. The cell viability was compared between the doxycycline-treated and the non-treated experiments for PC9 and A549 cells.

## Supplementary information

Sleeping Beauty Transposon-Mediated Asparaginase Gene Delivery by a Nanoparticle Platform

## Data Availability

The datasets generated during the current study are available from the corresponding author on reasonable request.

## References

[1] Yin Hao, Kanasty Rosemary L., Eltoukhy Ahmed A., Vegas Arturo J., Dorkin J. Robert, Anderson Daniel G. (2014). Non-viral vectors for gene-based therapy. Nature Reviews Genetics.

[2] Schatzlein AG (2001). Non-viral vectors in cancer gene therapy: principles and progress. Anti-Cancer Drug.

[3] Ivics Z, Hackett PB, Plasterk RH, Izsvak Z (1997). Molecular reconstruction of Sleeping beauty, a Tc1-like transposon from fish, and its transposition in human cells. Cell.

[4] Ohlfest JR, Lobitz PD, Perkinson SG, Largaespada DA (2004). Integration and long-term expression in xenografted human glioblastoma cells using a plasmid-based transposon system. Mol. Ther..

[5] Aronovich EL, McIvor RS, Hackett PB (2011). The Sleeping Beauty transposon system: a non-viral vector for gene therapy. Hum. Mol. Genet..

[6] Zhang Y, Satterlee A, Huang L (2012). *In Vivo* Gene Delivery by Nonviral Vectors: Overcoming Hurdles?. Mol. Ther..

[7] Al-Dosari MS, Gao X (2009). Nonviral Gene Delivery: Principle, Limitations, and Recent Progress. Aaps J..

[8] Wang K, Kievit FM, Zhang MQ (2016). Nanoparticles for cancer gene therapy: Recent advances, challenges, and strategies. Pharmacol. Res..

[9] Holcenberg JS (1981). Enzyme therapy of cancer, future studies. Cancer trea. Rep..

[10] Jackson JA, Halvorson HR, Furlong JW, Lucast KD, Shore JD (1979). A new extracorporeal reactor-dialyzer for enzyme therapy using immobilized L-asparaginase. J. Pharmacol. Exp. Ther..

[11] Balasubramanian MN, Butterworth EA, Kilberg MS (2013). Asparagine synthetase: regulation by cell stress and involvement in tumor biology. Am. J. Physiol.-Endoc. M..

[12] DeBerardinis, R. J. & Chandel, N. S. Fundamentals of cancer metabolism. *Sci*. *Adv*. **2** (2016). 10.1126/sciadv.1600200 PMC4928883 27386546

[13] Zhang J (2014). Asparagine plays a critical role in regulating cellular adaptation to glutamine depletion. Mol. cell.

[14] Krall AS, Xu S, Graeber TG, Braas D, Christofk HR (2016). Asparagine promotes cancer cell proliferation through use as an amino acid exchange factor. Nat. Commun..

[15] Alfadhel M (2015). Asparagine Synthetase Deficiency: New Inborn Errors of Metabolism. JIMD Rep..

[16] Li H (2016). Knockdown of asparagine synthetase by RNAi suppresses cell growth in human melanoma cells and epidermoid carcinoma cells. Biotechnol. Appl. Biochem..

[17] Panosyan EH (2014). Asparagine Depletion Potentiates the Cytotoxic Effect of Chemotherapy against Brain Tumors. Mol. Cancer Res..

[18] Cantor JR, Panayiotou V, Agnello G, Georgiou G, Stones EM (2012). Engineering Reduced-Immunogenicity Enzymes for Amino Acid Depletion Therapy in Cancer. Method Enzymol..

[19] Avramis VI, Tiwari PN (2006). Asparaginase (native ASNase or pegylated ASNase) in the treatment of acute lymphoblastic leukemia. Int. J. Nanomedicine.

[20] van den Berg H (2011). Asparaginase revisited. Leuk. lymphoma.

[21] Avramis VI (2012). Asparaginases: biochemical pharmacology and modes of drug resistance. Anticancer Res..

[22] Zhang B (2016). Targeting asparagine and autophagy for pulmonary adenocarcinoma therapy. Appl. Microbiol. Biotechnol..

[23] Chan WKLPL (2014). The glutaminase activity of L-asparaginase is not required for anticancer activity against ASNS-negative cells. Blood.

[24] Parmentier JHMMTE, Scotti C, Avramis VI, Mittelman SD (2015). Glutaminase Activity Determines Cytotoxicity of L-Asparaginases on Most Leukemia Cell Lines. Leuk. Res..

[25] Wang Z, Shi X, Li Y, Fan J, Zeng X, Xian Z, Wang Z, Sun Y, Wang S, Song P, Zhao S, Hu H, Ju D (2014). Blocking autophagy enhanced cytotoxicity induced by recombinant human arginase in triple-negative breast cancer cells. Cell Death & Disease.

[26] Yeh TH (2016). Selective Intracellular Delivery of Recombinant Arginine Deiminase (ADI) Using pH-Sensitive Cell Penetrating Peptides To Overcome ADI Resistance in Hypoxic Breast. Cancer Cells. Mol. Pharmaceut..

[27] Savaraj N (2010). Arginine deprivation, autophagy, apoptosis (AAA) for the treatment of melanoma. Curr. Mol. Med..

[28] Narta UK, Kanwar SS, Azmi W (2007). Pharmacological and clinical evaluation of L-asparaginase in the treatment of leukemia. Crit. Rev. Oncol. Hemat..

[29] Fung, M. K. L. & Chan, G. C. F. Drug-induced amino acid deprivation as strategy for cancer therapy. *J*. *Hematol*. *Oncol*. **10** (2017). 10.1186/s13045-017-0509-9 PMC5530962 28750681

[30] Smith TT (2017). *In situ* programming of leukaemia-specific T cells using synthetic DNA nanocarriers. Nat. Nanotechnol..

[31] Yant SR (2000). Somatic integration and long-term transgene expression in normal and haemophilic mice using a DNA transposon system. Nat. Genet..

[32] Horie K (2001). Efficient chromosomal transposition of a Tc1/mariner-like transposon Sleeping Beauty in mice. P. Natl. Acad. Sci. USA.

[33] Horie K (2003). Characterization of Sleeping Beauty transposition and its application to genetic screening in mice. Mol. Cell Biol..

[34] Clark KJ, Geurts AM, Bell JB, Hackett PB (2004). Transposon vectors for gene-trap insertional mutagenesis in vertebrates. Genesis.

[35] Converse AD (2004). Counterselection and co-delivery of transposon and transposase functions for Sleeping Beauty-mediated transposition in cultured mammalian cells. Bioscience Rep..

[36] Ma K (2017). Targeted delivery of *in situ* PCR-amplified Sleeping Beauty transposon genes to cancer cells with lipid-based nanoparticle-like protocells. Biomaterials.

[37] Chen W, Tsai P-H, Hung Y, Chiou S-H, Mou C-Y (2013). Nonviral Cell Labeling and Differentiation Agent for Induced Pluripotent Stem Cells Based on Mesoporous Silica Nanoparticles. Acs Nano.

[38] Chang JH, Tsai PH, Chen W, Chiou SH, Mou CY (2017). Dual delivery of siRNA and plasmid DNA using mesoporous silica nanoparticles to differentiate induced pluripotent stem cells into dopaminergic neurons. J. Mater. Chem. B.

[39] Chang FP, Chen YP, Mou CY (2014). Intracellular Implantation of Enzymes in Hollow Silica Nanospheres for Protein Therapy: Cascade System of Superoxide Dismutase and Catalase. Small.

[40] Seregin SS, Amalfitano A (2011). Gene Therapy for Lysosomal Storage Diseases: Progress, Challenges and Future Prospects. Curr. Pharm. Design.

[41] Schultz ML, Tecedor L, Chang M, Davidson BL (2011). Clarifying lysosomal storage diseases. Trends Neurosci..

