# Supplementary material for: Sleeping Beauty Transposon-Mediated Asparaginase Gene Delivery by a Nanoparticle Platform
Source: Sci Rep. 2019 Aug 7;9:11457. doi: 10.1038/s41598-019-47927-6 (PMC6686048; doi:10.1038/s41598-019-47927-6)
Supplement: Supplementary file 1 — Sleeping Beauty Transposon-Mediated Asparaginase Gene Delivery by a Nanoparticle Platform [file 41598_2019_47927_MOESM1_ESM.pdf]

SUPPORTING INFORMATION

Sleeping Beauty Transposon-Mediated Asparaginase Gene Delivery by a Nanoparticle Platform

Jen-Hsuan Chang[a], Yun Mou[b] and Chung-Yuan Mou[a,c*]

[a] Center for Condensed Matter Sciences, and Department of Chemistry, National Taiwan University, Taipei, Taiwan 10617

[b] Institute of Biomedical Sciences, Academia Sinica, Taipei, Taiwan 11529

[c]Graduate Institute of Nanomedicine and Medical Engineering, Taipei Medical University, No. 250, Wu Xinyi Street, Taipei 11031, Taiwan.

List of Tables and Figures:

Table S1. The primer sequence used in molecular cloning……………………………………………1

Table S2. The zeta potential and DLS of PEI-adsorbed MSN (MP) and T+PEI-adsorbed MSN (MPT), T: pSB-ASNase+SB100……………………………1

Figure S1. The PCR product of the asparaginase gene is amplified from Escherichia coli………………………………………………2

Figure S2. The thermogravimetric analysis of MSN-NH_2 (M) and PEI-adsorbed MSN (MP)...............…………3

Figure S3. The time-course confocal microscopy analysis showed that FMSN escaped from endosome in PC9 cells............…………4

Figure S4. The transfection efficiency is analyzed by the flow cytometry............…………5

Figure S5. The qPCR quantification of asparaginase fold induction in MPT-A549 and MPT-PC9 cell lines………………6

Figure S6. The IC50 analysis of asparaginase are treated on the (a): PC9 cells and (b): A549 cells ……………7

Figure S7. Different concentrations of doxycycline were treated on PC9 or A549 cells.…………8

Figure S8. The asparaginase-integrated stable cell clones of PC9 and A549…....9

Figure S9. Comparison of the cell viability between the parental PC9 and the ASNase-carried PC9 after 24 hours of doxycycline induction in RPMI (left) and DMEM (right)...…...10

Table S1. The primer sequence used in molecular cloning

Gene	Primer sequence (5' → 3')
**pSB-ASNase**	**F:** ATACGACTCACTATAGGGACTAGTGCCACCATG GAGTTTTTCAAAAAGACGGCACTTG<br>**R:** ATGTCTGGATCCCCGCGGTTAGGGCCCATTAGTA CTGATTGAAGATCTGCTGGATCTG

Table S2. The zeta potential and DLS of PEI-adsorbed MSN (MP) and T+PEI-adsorbed MSN (MPT), T: pSB-ASNase+SB100

	Zeta potential (mV, H_2O, pH=7.4)	DLS size (nm, H_2O)
**MP (PEI: 2.25μg)**	+32.7	186.4
**MPT (PEI: 2.25μg)**	+29.6	208.0
**MP (PEI: 4.5μg)**	+40.9	182.7
**MPT (PEI: 4.5μg)**	+22.5	447.1

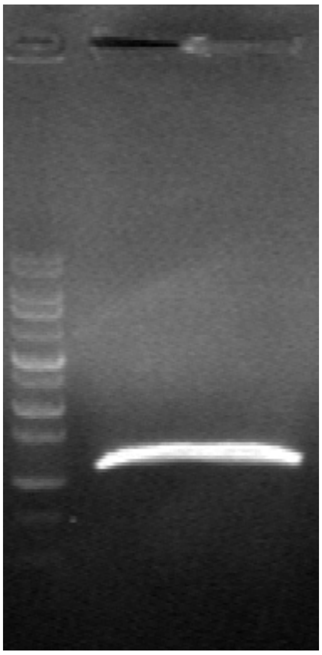

Figure S1. The PCR product of the asparaginase gene is amplified from *Escherichia coli*. The sizes of the ASNase gene is 1048 bp.

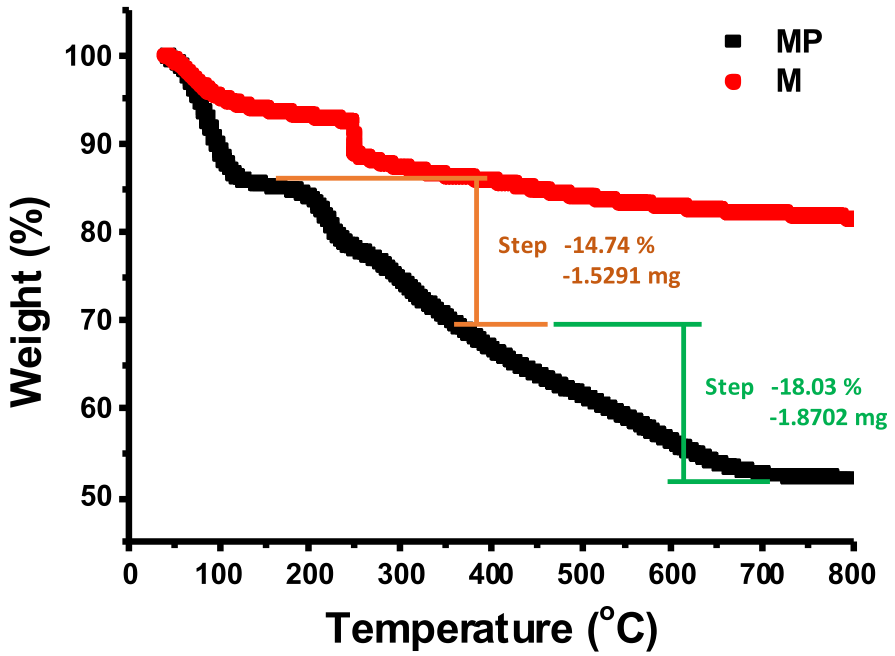

Figure S2. The thermogravimetric analysis of MSN-NH_2 (M) and PEI-adsorbed MSN (MP). The differential thermal analysis showed that the first weight drop from 200-400 °C is the loss of amination (14.74%). The second weight drop from 400-600 °C is the loss of PEI.

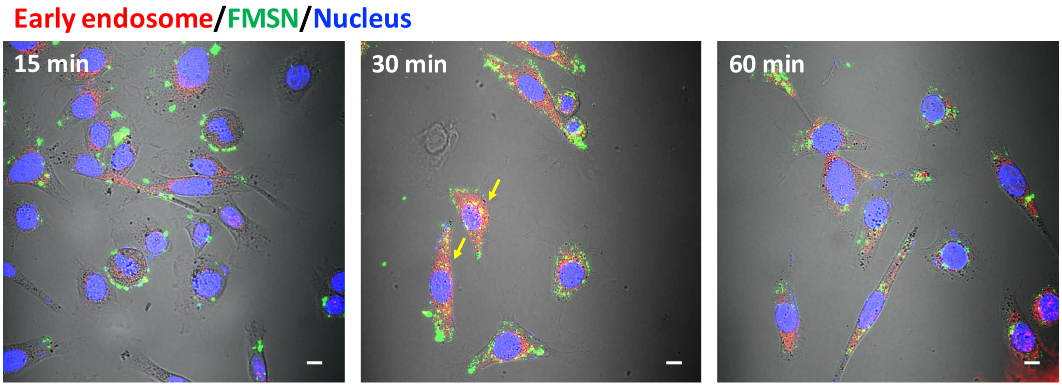

Figure S3. The time-course confocal microscopy analysis showed that FMSN escaped from endosome in PC9 cells. Left: for 15 min; Middle: for 30 min; Right: for 60 min. FITC-conjugated MSN-NH_2 (FMSN, green), early endosome marker EEA1 (red), DAPI (blue), and the bright field of images were merged together. The PC9 cells was incubated with 25 µg/mL FMSN. Scale bars are 10 µm.

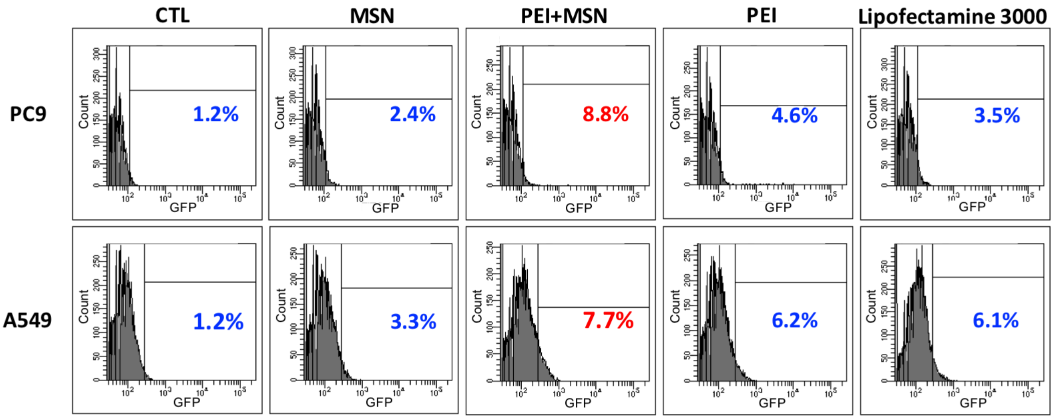

Figure S4. The PEI-MSN particles showed a superior transfection efficiency to other common transfection reagents in PC9 or A549 cells, including PEI and Lipofectamine 3000. The *Sleeping Beauty* plasmids (pSB-ASNase and SB100) were co-transfected into PC9 or A549 cells. After 48 hours, the transfection efficiency was determined by GFP expression using flow cytometry.

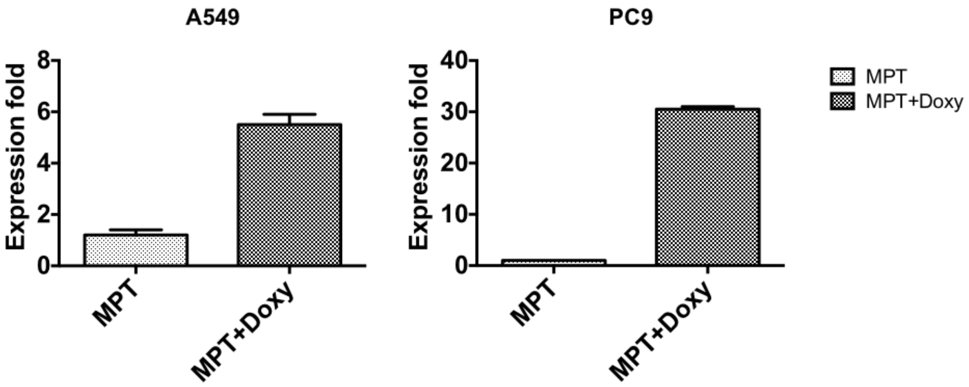

Figure S5. The qPCR quantification of asparaginase fold induction in MPT-A549 and MPT-PC9 cell lines. The before-induction value was normalized to 1.

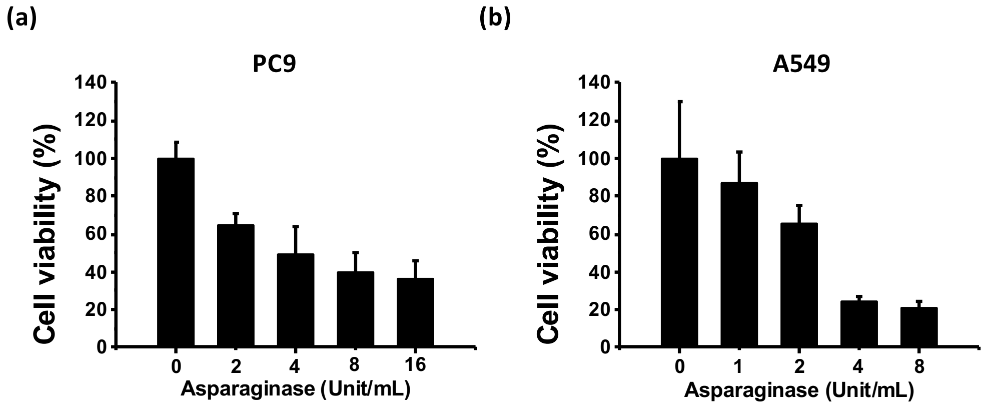

Figure S6. PC9 and A549 cells are sensitive to the asparagine depletion by adding L-asparaginase in the growth medium. (a) PC9 cells, and (b) A549 cells. One unit of L-asparaginase is defined as that amount of enzyme required to generate 1 μmol of ammonia/min at pH 7.3 and 37°C.

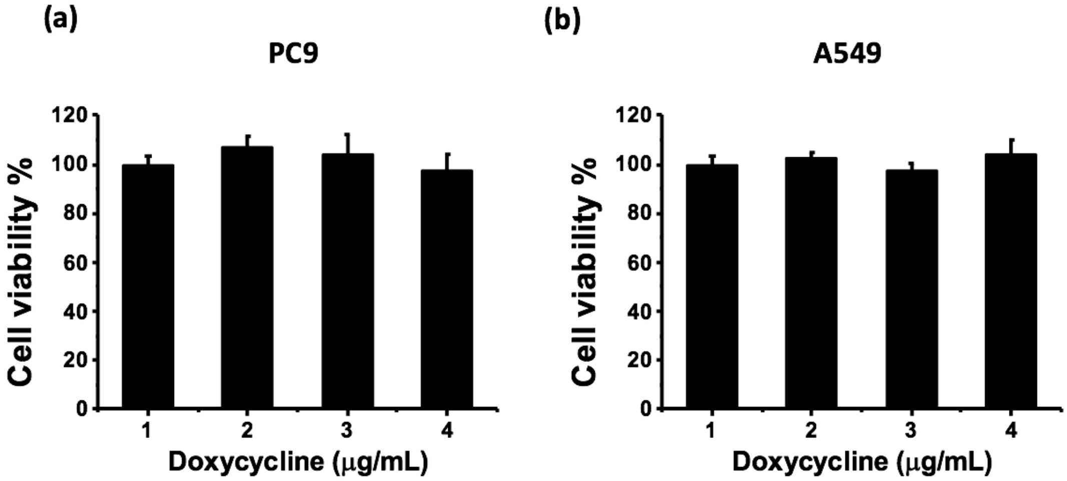

Figure S7. The doxycycline treatment is not cytotoxic to the PC9 and A549 cells. Different concentrations of doxycycline were treated on PC9 or A549 cells. The cell viability was assayed after 48 hours.

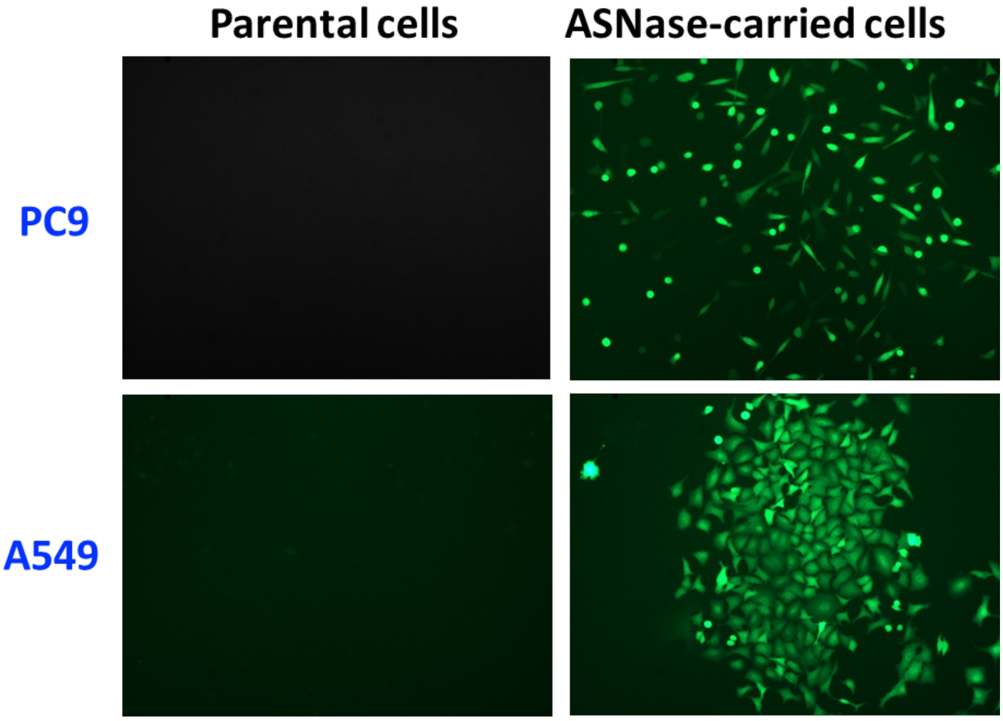

Figure S8. The asparaginase-integrated stable cell clones of PC9 and A549 are successfully generated. The green fluorescent protein (GFP) was stably expressed in the cell clones observed by florescence microscope. MPT: T+PEI-adsorbed MSN. T: pSB-ASNase+SB100.

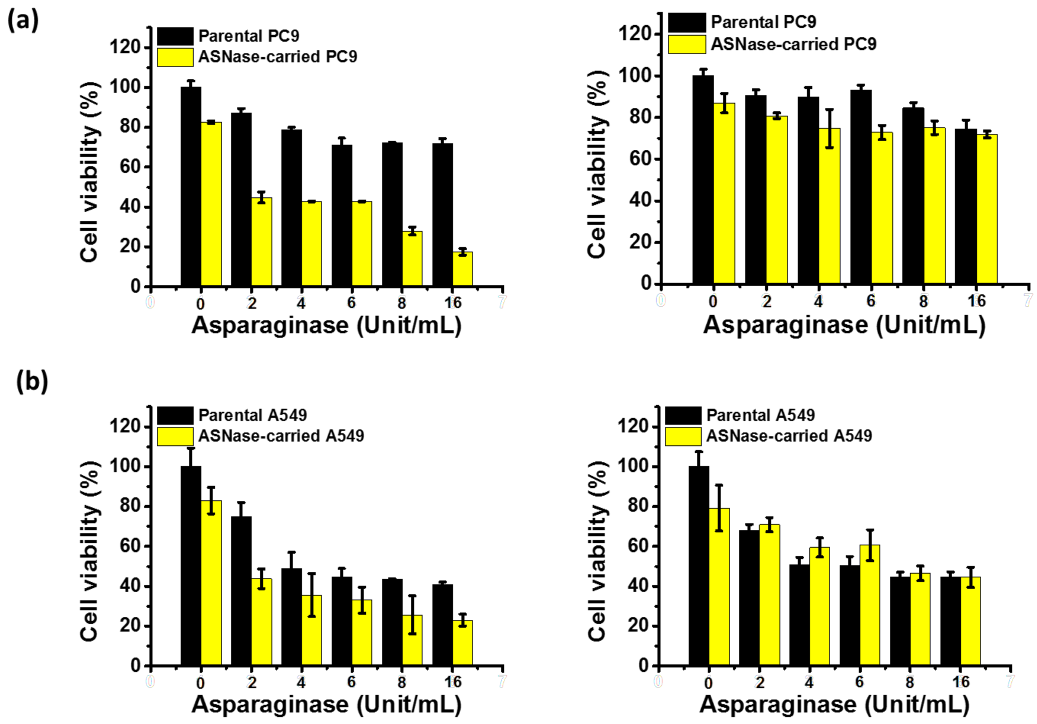

Figure S9. The ASNase-carried PC9 and A549 are hyper-sensitive to the exogenous asparaginase treatment. (a) Comparison of the cell viability between the parental PC9 and the ASNase-carried PC9 after 24 hours of doxycycline induction in RPMI (left) and DMEM (right). (b) Comparison of the cell viability between the parental A549 and the ASNase-carried A549 after 48 hours of doxycycline induction in RPMI (left) and DMEM (right).
